# Antimicrobial and cytotoxic activities of flavonoid and phenolics extracted from *Sepia pharaonis* ink (Mollusca: Cephalopoda)

**DOI:** 10.1186/s12896-024-00880-3

**Published:** 2024-08-12

**Authors:** Asmaa R. Abdel-Malek, Alaa Y. Moustafa, Shimaa H. Salem

**Affiliations:** 1 Zoology and Entomology Department, Faculty of Science, Assiut University, Assiut, 71526 Egypt; 2 Zoology Department, Faculty of Science, Sohag University, Sohag, 82524 Egypt; 3 Botany and Microbiology Department, Faculty of Science, Assiut University, Assiut, 71526 Egypt

**Keywords:** Flavonoids, Red Sea, Cytotoxicity, Cephalopods, Antimicrobial activity, Seagrass, Flow cytometry

## Abstract

**Background:**

Several studies have been reported previously on the bioactivities of different extracts of marine molluscs. Therefore, we decided to evaluate the cytotoxic and antimicrobial activities of *S. pharaonis* ink as a highly populated species in the Red Sea. We extracted the flavonoids from the ink and analyzed their composition. Then we evaluated systematically the cytotoxic and antimicrobial properties of this extract. A pharmacokinetic study was also conducted using SwissADME to assess the potential of the identified flavonoids and phenolic compounds from the ink extract to be orally active drug candidates.

**Results:**

Cytotoxic activity was evaluated against 5 cell lines (MCF7, Hep G2, A549, and Caco2) at different concentrations (0.4 µg/mL, 1.6 µg/mL, 6.3 µg/mL, 25 µg/mL, 100 µg/mL). The viability of examined cells was reduced by the extract in a concentration-dependent manner. The highest cytotoxic effect of the extract was recorded against A549 and Hep G2 cancer cell lines cells with IC_50 =_ 2.873 and 7.1 µg/mL respectively. The mechanistic analysis by flow cytometry of this extract on cell cycle progression and apoptosis induction indicated that the extract arrests the cell cycle at the S phase in Hep G2 and MCF7, while in A549 cell arrest was recorded at G1 phase. However, it causes G1 and S phase arrest in Caco2 cancer cell line. Our data showed that the extract has significant antimicrobial activity against all tested human microbial pathogens. However, the best inhibitory effect was observed against *Candida albicans* ATCC 10,221 with a minimum inhibitory concentration (MIC) of 1.95 µg/mL. Pharmacokinetic analysis using SwissADME showed that most flavonoids and phenolics compounds have high drug similarity as they satisfy Lipinski’s criteria and have WLOGP values below 5.88 and TPSA below 131.6 Å^2^.

**Conclusion:**

*S. pharaonis* ink ethanolic extract showed a promising cytotoxic potency against various cell lines and a remarkable antimicrobial action against different pathogenic microbial strains. *S. pharaonis* ink is a novel source of important flavonoids that could be used in the future in different applications as a naturally safe and feasible alternative of synthetic drugs.

**Supplementary Information:**

The online version contains supplementary material available at 10.1186/s12896-024-00880-3.

## Introduction

Cephalopods including the cuttlefish *Sepia pharaonis* are the most advanced group in phylum Mollusca [1]. They provide a wide range of human resources, including food, shells, dyes, and medicines, so they have received special attention and become a natural resource of economic importance [2–5]. These marine molluscs have several defense mechanisms against their predators such as the ability to change color, shape, and the most famous escaping behavior “inking” [6–10]. The ink defense behavior of Cephalopods depends on the activity of a highly specialized gland known as “the ink sac” which is responsible for the production of black ink. In addition to the funnel organ, which acts as a mucus-producing gland. This secretion passes through the gland aperture, accumulates in the ink sac cavity, and ejects on demand [11–14]. The ink is traditionally used by humans for numerous commercial and practical purposes such as food, art, and several medical applications [2–5]. Chemically, this ink consists mainly of melanin granules [14, 15] in the form of eumelanin which is a polymer of 5,6-dihydroxyindole and 5,6-dihodroxyindole-2-carboxylic acid derived from tyrosine. The ink also contains proteins, glycosaminoglycans, lipids, amino acids, melanogenic enzymes including tyrosinase, and some heavy metals like cadmium and copper [11, 14, 16, 17]. Several medicinal and therapeutic effects of cephalopod ink have been reported involving; anti-cancer, antimicrobial, antioxidant, antiretroviral, Anti-ulceration, Anti-Hypertensive, and Hepatoprotective actions [16, 18–25]. The anti-cancer effects of squid and cuttlefish ink occur through the initiation of apoptosis and are affiliated with different chemicals of ink [10, 15, 19, 26]. Antimicrobial properties of cephalopods ink have been determined [27–32]. Flavonoids are a group of natural products (plant secondary metabolites) with variable phenolic structures [33]. They are synthesized via the phenylpropanoid pathway. Flavonoids have 15 carbon atoms and share a common C6-C3-C6 skeleton, with two benzene rings linked through a heterocyclic pyrane or a pyrone ring. They are classified into various subgroups according to the chemical structure, degree of unsaturation, and oxidation of carbon ring including chalcones, anthocyanins, flavones, anthoxanthins (flavonols, flavanones), and isoflavonoids [34, 35]. Flavonoids have been shown to possess anti-inflammatory, antioxidant, antibacterial, antiviral, hepatoprotective, antiallergic, antithrombotic, anticarcinogenic Alzheimer’s disease (AD), atherosclerosis and immunomodulator activities in some in vitro and animal model studies [36–38]. Flavonoids have been only recorded in marine sea grass e.g., *Enhalus acoroides* [39] genus Zostera [40–42]. No previous articles recorded flavonoids in marine animals. Interestingly, the sea grass environment acts as a feeding, reproduction, and nursery area for cuttlefishes. In addition, it provides shelter from predation [43]. The goal of the present study is to evaluate the cytotoxic and antimicrobial activities of flavonoid extract, for the first time, from the ink of cuttlefish *Sepia pharaonis* Ehrenberg, 1,831 (pharaoh cuttlefish) which is commonly distributed on the Egyptian coast of the Red Sea.

## Materials and methods

### Chemicals and reagents

In vitro toxicology assay kit, MTT based (No. TOX1, Sigma-Aldrich, USA). Propidium iodide flow cytometry kit (No. ab 139,418, abcam, US). Annexin V-FITC Apoptosis Detection Kit (No. K101-25, Biovision, USA). Dulbecco’s modified Eagle’s medium (DMEM; Invitrogen/Life Technologies).

### Collection and identification of the cuttlefish samples

The cuttlefish *S. pharaonis* were collected by a fisherman from the coastal water of Hurghada in the Red Sea governorate, Egypt. Upon capture, they were immediately frozen, placed in ice tanks, and transported into the laboratory. Subsequently, they underwent taxonomic identification according to Riad et al. [1], after which they were dissected without anesthesia. The ventral mantle was carefully opened, the ink sac was removed, squeezed and the ink was collected in a sterilized falcon tube and stored at -20 °C for further analysis.

### Preparation of ***S. pharaonis***ink extract (IE)

The collected ink was centrifuged and then 10 ml of the supernatant (melanin-free ink) was extracted with 70% ethanol by stirring (400 rpm) at room temperature for 24 h [44]. The extract was dried at 40 °C under reduced pressure using a rotatory evaporator (Heidolph, Type VV1, No. 51,111, Germany). The obtained dried powder was stored at -20 °C until further use. For chromatographic characterization, the extract was dissolved in 80% methanol, while the extract was dissolved in DMSO for biological evaluation.

### HPLC analysis of flavonoid and phenolic in the IE

The analysis of flavonoid and phenolic compounds in the *S. pharaonis* ink extract was conducted using HPLC equipment (Agilent 1260 series). Separation of compounds was achieved using an Eclipse C18 column (250 mm x 4.6 mm I.D; particle size 5 μm). The column temperature was maintained at 40 °C, and an injection volume of 5 µl was employed for each sample solution. Compounds were separated employing a gradient mobile phase consisting of water (A) and 0.05% trifluoroacetic acid in acetonitrile (B), with a flow rate of 0.9 ml/min. The mobile phase gradient profile was programmed as follows: 0 min (82% A); 0–5 min (80% A); 5–8 min (60% A); 8–12 min (60% A); 12–15 min (82% A); 15–16 min (82% A); and 16–20 min (82% A). Detection of resolved compounds was conducted using a multi-wavelength detector set to monitor at 280 nm. Compound identification was carried out based on available standards of phenolic and flavonoid compounds.

## Evaluation of biological activities of the extract

### Cytotoxic activity

#### Cell lines

The extract was examined on 4 cancer cell lines: the breast cancer cell MCF7 (HTB-22), liver cancer cell Hep G2 (HB-8065), lung cancer cell A549 (CCL-185), Colon cancer cell Caco2 (HTB-37), and a normal cell line WI38 (CCL-75) which were obtained from American Type Culture Collection. The experiment was carried out in Vacsera-El-Doky- Cairo- Egypt.

### Cell culture and treatments

Cells were cultured in DMEM supplemented with 10% FBS (Hyclone), 10 µg/mL of insulin (Sigma), and 1% penicillin-streptomycin. Plate cells (cells density 1.2–1.8 × 10,000 cells/well) in a volume of 100 µl complete growth medium + 100 µl of the tested compound per well in a 96-well plate were incubated for 24 h before the MTT assay which was used to measure the effect of the extract on the examined cell lines. Control represented by untreated wells and the treatments include 5 concentrations of the extract (0.4 µg/mL, 1.6 µg/mL, 6.3 µg/mL, 25 µg/mL, 100 µg/mL) and compared with a standard cytotoxic compound, staurosporine. After treatment, cells were incubated for 48 h at 37^0^ C and then preceded for MTT assay.

### MTT assay and the cell viability

MTT was added to the culture in an amount equal to 10% of the medium volume and incubated for 2–4 h depending on cell type. The resulting formazan crystals were dissolved by adding an amount of MTT Solubilization Solution [M-8910] equal to the original culture medium volume. Spectrophotometrically the absorbance was measured at a wavelength of 570 nm. The percentage of cell viability and Cytotoxicity were calculated as follows:

Cell viability (%) = (Absorbance of the sample/Absorbance of control) x100.

Cytotoxicity (%) = 100 - Percentage of Viability.

### Analysis of the cell cycle

The MCF7, Hep G2, A549, and Caco2 cancer cell lines were examined using the extract’s pre-calculated IC_50_. Pipette the cells gently up and down to resuspend them. For five minutes, pellet the cells at 500 x g. Without disturbing the particle, carefully aspirate the supernatant. Rinse the cells in 1 mL of 1X PBS, gently resuspending them. Once more, pellet the cells for five minutes at 500 x g, then carefully extract the supernatant. In 200 µL of 1X Propidium Iodide + RNase Staining Solution, gently resuspend the cell pellet. For twenty to thirty minutes, incubate at 37ºC in the dark. Set up tubes for flow cytometry analysis by placing them on ice while they are still dark. Pipette up and down to gently resuspend any cells that settled throughout the incubation. If needed, eliminate cell clumps by passing cells through a suitable filter (not supplied). Use a flow cytometer to run samples: To keep trash and cell aggregates out, set the proper FSC vs. SSC gates. Gather the fluorescence of propidium iodide in FL2.

### Analysis of necrosis and apoptosis using flow cytometry

By labeling the investigated cancer cells with annexin V-fluorescein isothiocyanate (FITC) or propidium iodide (PI) in accordance with the Annexin V-FITC apoptosis detection kit, apoptosis and necrosis were assessed. In short, the cultivated cells underwent multiple PBS washes after being trypsinized. Centrifugation was used to harvest 1–5 × 10^5^ cells, which were then suspended in 500 µl of 1x binding buffer. Following this, 5 µl each of Annexin V-FITC and propidium iodide were added, and the suspension was incubated for 5 minutes at room temperature in the dark, prior to flow cytometry analysis.

### Antimicrobial activity assay

#### Microbial strains used

Four bacterial strains including [*Escherichia coli* ATCC 8739, *Pseudomonas aeruginosa* ATCC 90,274, *Bacillus subtilis* ATCC 6633, and *Staphylococcus aureus* ATCC 6538], and two fungal strains [*Candida albicans* ATCC 10,221, and a clinical isolate of *Aspergillus niger* obtained from Assiut University Mycological Centre (AUMC)] were used in this experiment.

### Agar well diffusion assay

The antimicrobial assay of crude ink extract was evaluated by agar well diffusion method in Luria-Bertani (LB) plates [45]. Initially, the actively growing bacterial broth cultures were adjusted to a concentration of 0.5 McFarland standard (equivalent to 1 × 10^8^ cfu/ml), while the fungal inoculum was prepared at a concentration of 10^6^ spore/ml. After inoculum preparation, one milliliter of the balanced inoculum was streaked on the sterile LB agar and Sabouraud dextrose agar (SDA) plates. On each plate, wells with a diameter of 8 mm were punched using a sterile agar borer. Then, 100 µL of the *S. pharaonis* ink extract (at a concentration of 3 mg/mL) was added directly into the corresponding wells, and the plates were transferred into an incubator. Gentamicin (10 mg/mL) for all bacterial strains and fluconazole (10 mg/mL) for fungal stains were used as a positive control. Negative control was 10% DMSO. The experiment was performed in triplicate. Antimicrobial activity was assessed by measuring the average zone of inhibition against each test sample.

### Determination of MIC and MBC values of the IE

Generally, MIC value is defined as the lowest concentration of a given antimicrobial agent that completely inhibited microbial growth after a specified incubation period. In our study, MIC was evaluated using the broth microdilution method outlined by Ferraro [46]. Briefly, 2-fold serial dilutions of the ink extract were prepared in a 96-well microtiter plate to obtain concentration series ranging from 1000 to 1.95 µg/mL. A hundred microliters of microbial growth were added to each dilution in their respective wells. Negative control wells contained only broth while positive control wells consisted of microorganisms and broth. Plates were incubated at 35 ± 2 °C for 16 to 20 h. After that, the optical density (OD) values were recorded at 600 nm using a microplate reader.

To determine minimum bactericidal concentration (MBC), concentrations that exhibited complete inhibition were streaked onto agar plates and incubated under the same conditions. The absence of microbial growth on the agar surface at the lowest concentration of the ink extract was identified as MBC or MFC.

### ADME and drug-likeness analysis

To assess the potentiality of flavonoids and phenolics from IE to be good drug candidates, we conducted ADME analysis and evaluated drug-likeness parameters using the free web tool SwissADME (Absorption, Distribution, Metabolism, and Excretion) (http://www.swissadme.ch/) [47]. The chemical structure of the compounds was drawn using MarvinJs.

### Statistical analysis

Data were analyzed by using GRAPHPAD PRISM (version 8.0) software and the significant differences (at *P* < 0.05) were determined by one-way analysis of variance (ANOVA) with applying Tukey’s test for multiple comparisons.

## Results

### High-performance liquid chromatography (HPLC) analysis of the IE

**Fig. 1:**
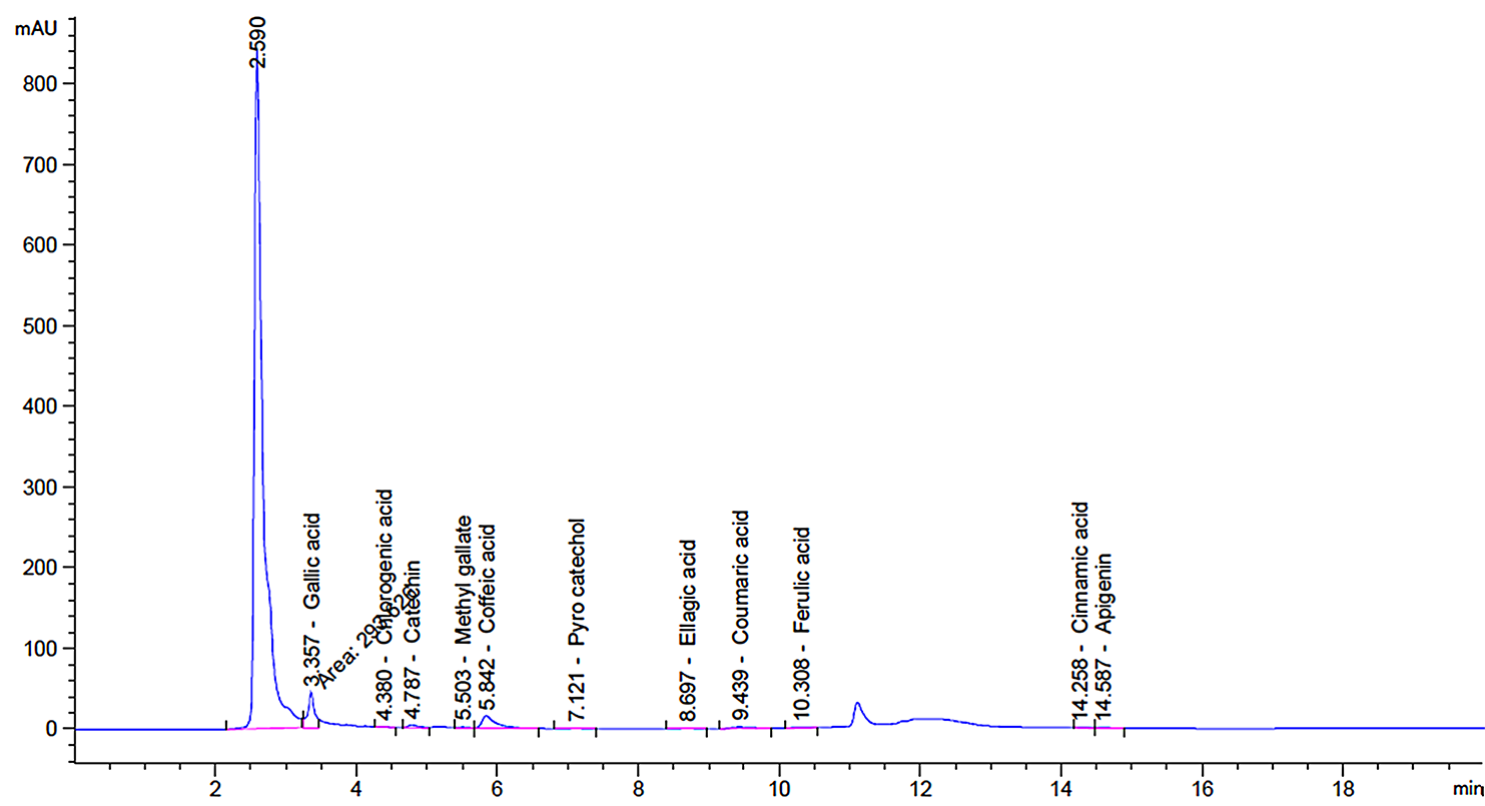
HPLC chromatogram of the extracted flavonoid and phenolics from the ink sac of *S*. *pharaonic*

The HPLC analysis of the ink extract unveiled the presence of various phenolic and flavonoid compounds, which were identified as gallic acid, chlorogenic acid, catechin, methyl gallate, caffeic acid, pyrocatechol, ellagic acid, coumaric acid, ferulic acid, cinnamic acid, and apigenin (as shown in Fig. 1). The chromatogram of standard flavonoids and phenolics used in this study is shown in Fig. S1. According to Table 1, the cuttlefish *S. pharaonis* exhibited high concentrations of gallic acid at 410.66 µg/g, followed by caffeic acid and catechin at concentrations of 258.53 µg/g and 155.79 µg/g, respectively.

**Table 1:** HPLC analysis of bioactive compounds in the ethanolic crude extract of *Sepia pharaonic* ink

Peak #	RT (min)	Compound name	Molecular formula	Molecular weight	Area (mAU*s)	Area (%)	Conc. (µg/g)
1	3.357	Gallic acid	C_7_H_6_O_5_	170.12	293.62610	3.7049	410.66
2	4.380	Chlorogenic acid	C_16_H_18_O_9_	354.31	5.22149	0.0659	13.00
3	4.787	Catechin	C_15_H_14_O_6_	290.27	37.99434	0.4794	155.79
4	5.503	Methyl gallate	C_8_H_8_O_5_	184.15	8.28224	0.1045	8.23
5	5.842	Caffeic acid	C_9_H_8_O_4_	180.16	204.44717	2.5796	258.53
6	7.121	Pyro catechol	C_6_H_6_O_2_	110.11	3.30806	0.0417	7.48
7	8.697	Ellagic acid	C_14_H_6_O_8_	302.19	5.79629	0.0731	14.41
8	9.439	Coumaric acid	C_9_H_8_O_3_	164.16	31.74083	0.4005	12.40
9	10.308	Ferulic acid	C_10_H_10_O_4_	194.18	10.98365	0.1386	11.49
10	14.258	Cinnamic acid	C_9_H_8_O_2_	148.16	2.14905	0.0271	0.76
11	14.587	Apigenin	C_15_H_10_O_5_	270.24	5.33759	0.0673	6.65

### Cytotoxic activity

**Fig. 2:**
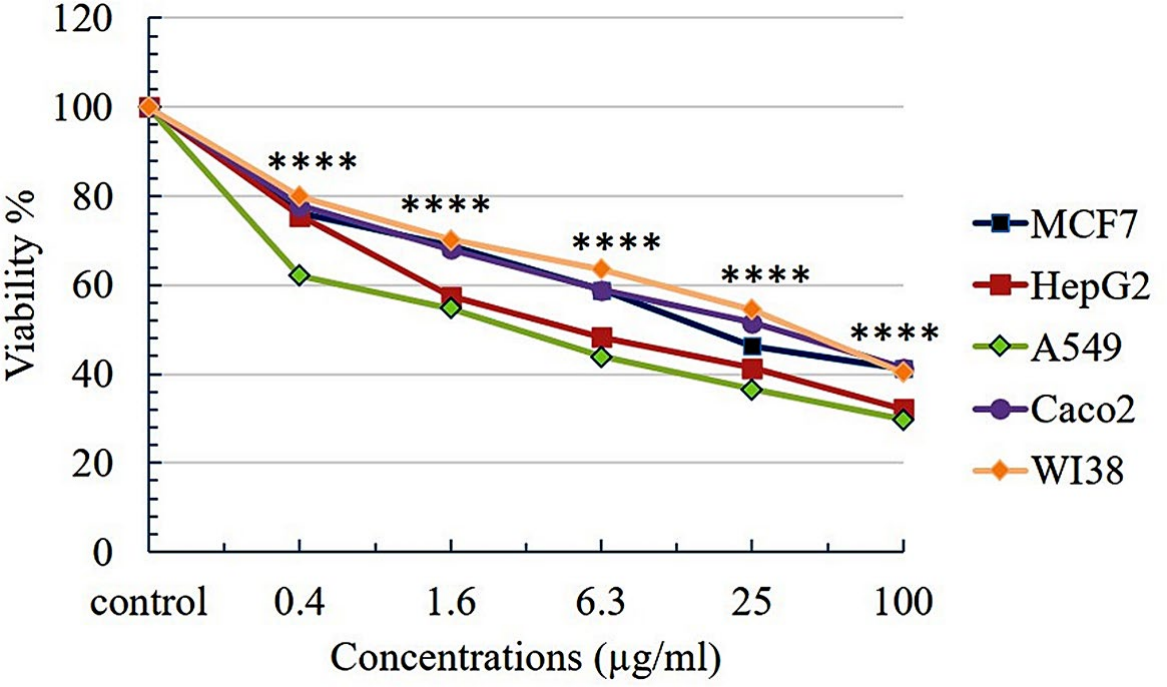
Cell viability (%) of all tested cell lines treated with different concentrations of the extract and after 24 h. Significant differences are based on the comparison between the control group and treated groups in each cell line. **** *P* > 0.0001

Cytotoxicity of flavonoids and phenolics-rich extract from the cuttlefish ink was carried out against MCF7, Hep G2, A549, Caco2, and WI38 by the MTT assay. The viability of these cells was reduced by the extract in a concentration-dependent manner (Fig. 2). The extract achieved its highest cytotoxic effect on A549 cancer cells with IC_50 =_ 2.873 µg/mL followed by Hep G2 (IC_50_ = 7.1 µg/mL), however MCF7 and Caco2 cancer cells demonstrated lower effect, with IC_50_ values of 21.62 µg/mL and 27.41 µg/mL, respectively. (Table 2).

**Table 2:** Cytotoxicity of ethanolic extract of *Sepia pharaonic* ink towards five different cancer cell lines

Treatment	IC_50_ values in µg/mL				
	MCF7	Hep G2	A549	Caco2	WI38
**Extract**	21.62 ± 1.16	7.101 ± 0.33	2.873 ± 0.12	27.41 ± 1.42	36.13 ± 2.16
**Staurosporine**	7.882 ± 0.42	6.331 ± 0.3	9.98 ± 0.43	5.47 ± 0.28	26.24 ± 1.57

### Effect of IE on cell cycle of the examined cancer cells

**Fig. 3:**
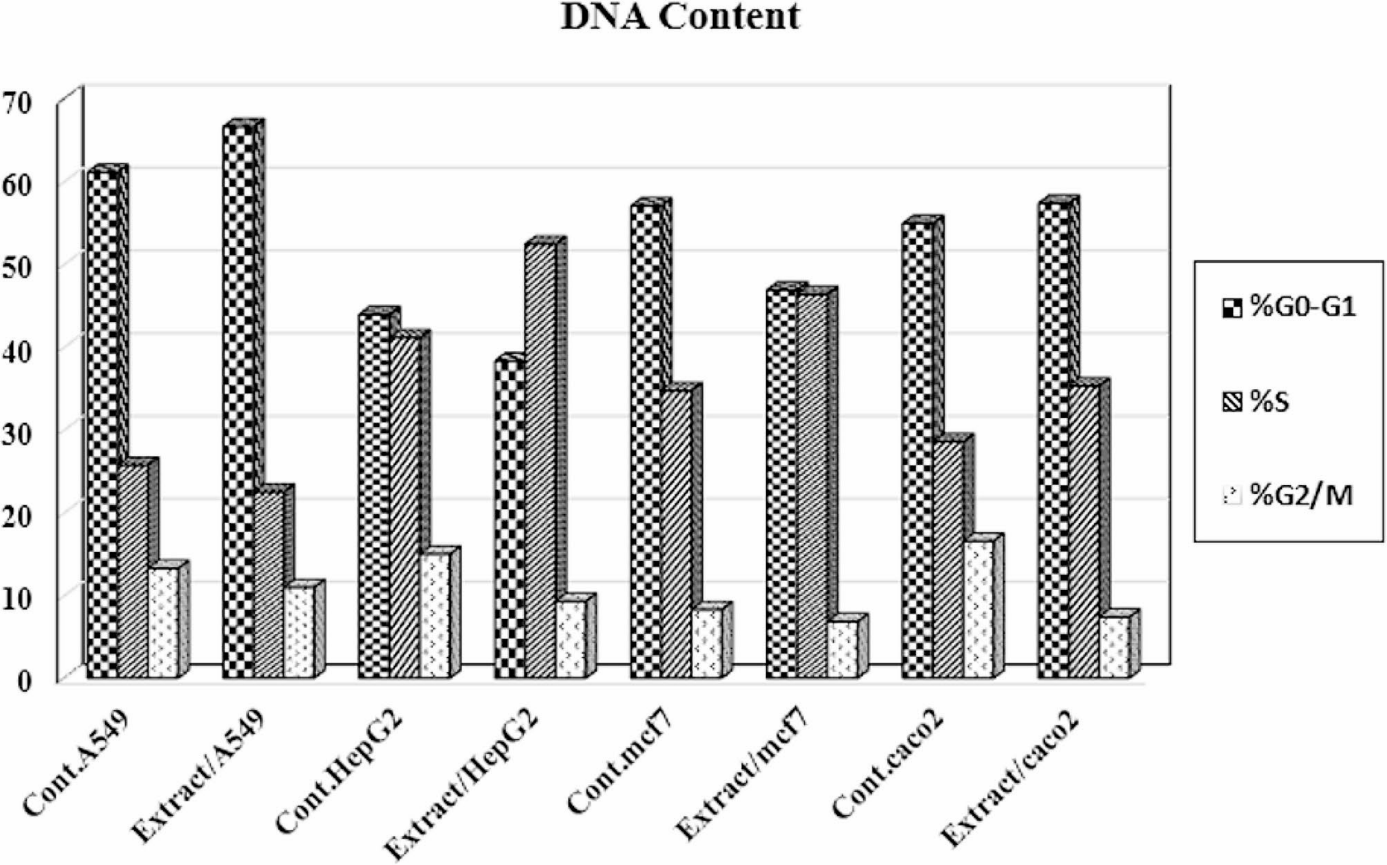
DNA contents of the examined cancer cells that were treated with the extract compared with the control

**Fig. 4:**
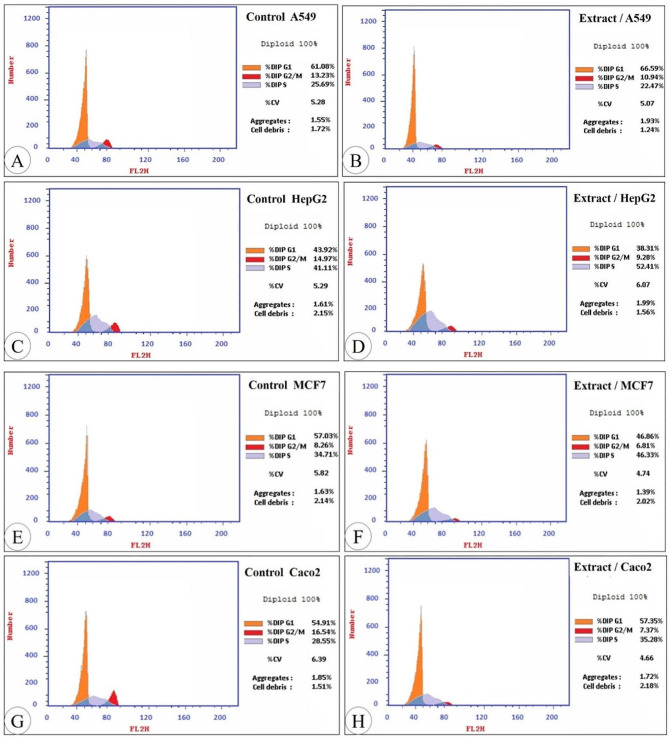
Cell cycle distribution of the lung cancer cells A549 (**A** and **B**), liver cancer cells Hep G2 (**C** and **D**), breast cancer cells MCF7 (**E** and **F**), and the colon cancer cells Caco2 (G and H) treated with the extract by propidium iodide staining using flow cytometry

The cancer cells (MCF7, Hep G2, A549, and Caco2) were treated with pre-calculated IC_50_ values for investigation of the intracellular mechanism of the extracted flavonoid action. After staining with propidium iodide (PI), cells were analyzed by flow cytometry for visualization of the changes in the DNA contents and cell cycle distribution. Cell growth arrest in the treated cancer cells was recorded at G_1_ phase in the case of A549. However, in the case of Hep G2, MCF7, and Caco2 it was recorded at S phase. (Figures 3 and 4).

### Apoptotic study of IE on the tested cancer cell lines

**Fig. 5:**
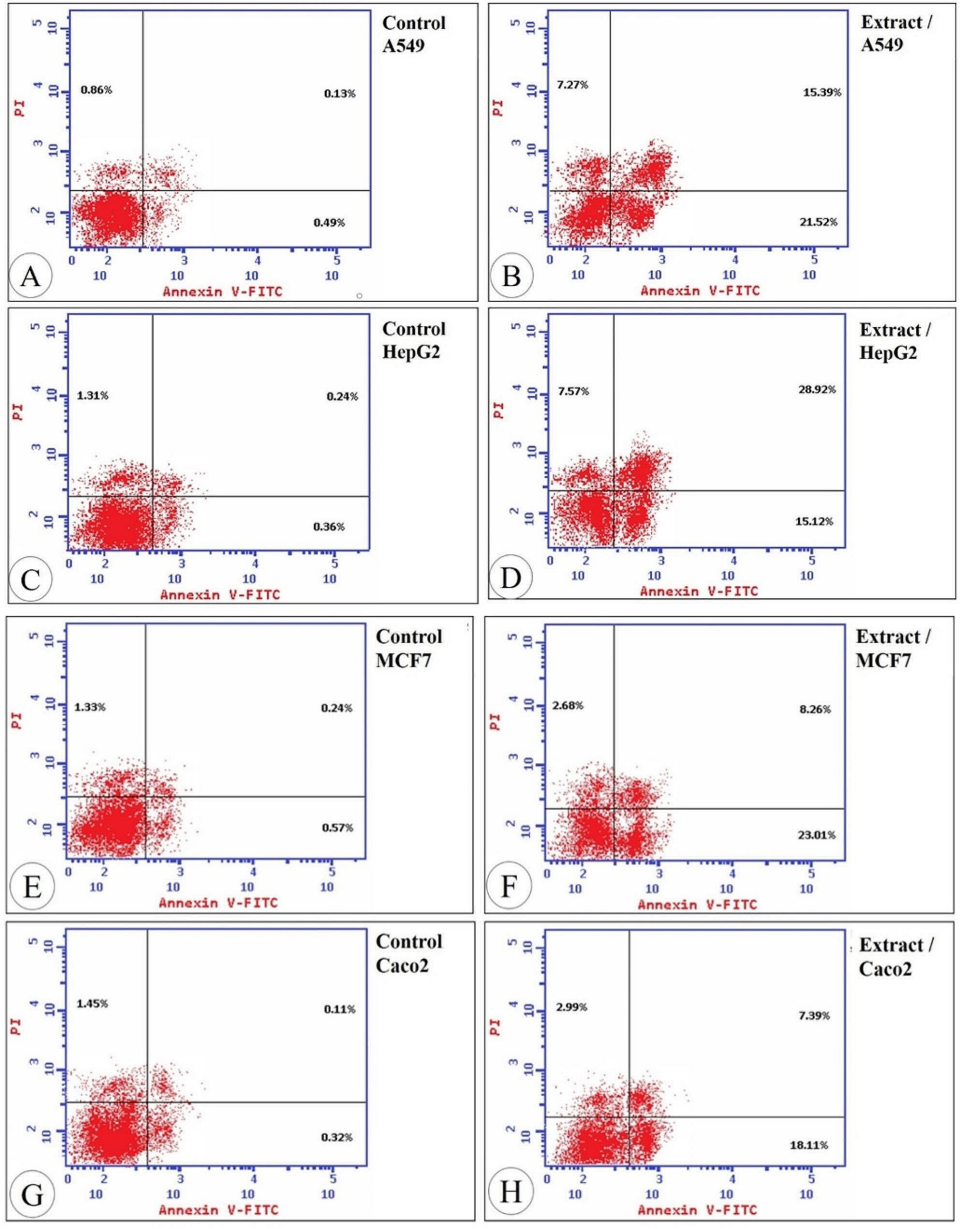
Apoptotic effect of the extract in the lung cancer cells A549 (**A** and **B**), liver cancer cells Hep G2 (**C** and **D**), breast cancer cells MCF7 (**E** and **F**), and the colon cancer cells Caco2 (**G** and **H**) treated with the extract by propidium iodide staining using flow cytometry

In cancer treatment, apoptosis is assumed to be a hallmark in the induction of cancer cell death. Flow cytometry was used for monitoring these cells after staining with Annexin V-FITC/PI. The highest percentage of apoptosis and necrosis was recorded in Hep G2 (51.61% and 7.57% respectively) as shown in Fig. 5; Table 3.

**Table 3:** The percentages of apoptotic and necrotic cells induced by ethanolic extract of *Sepia pharaonic* ink towards four different cancer cell lines

Cell type	A549			Hep G2			MCF7			Caco2	
Treatment	Control	Extract		Control	Extract		Control	Extract		Control	Extract
% All dead cells	1.48	44.18		1.91	51.61		2.14	33.95		1.88	28.49
% Early apoptotic cells	0.49	21.52		0.36	15.12		0.57	23.01		0.32	18.11
% Late apoptotic cells	0.13	15.39		0.24	28.92		0.24	8.26		0.11	7.39
% Necrotic cells	0.86	7.27		1.31	7.57		1.33	2.68		1.45	2.99

### Antimicrobial activity of IE

**Fig. 6:**
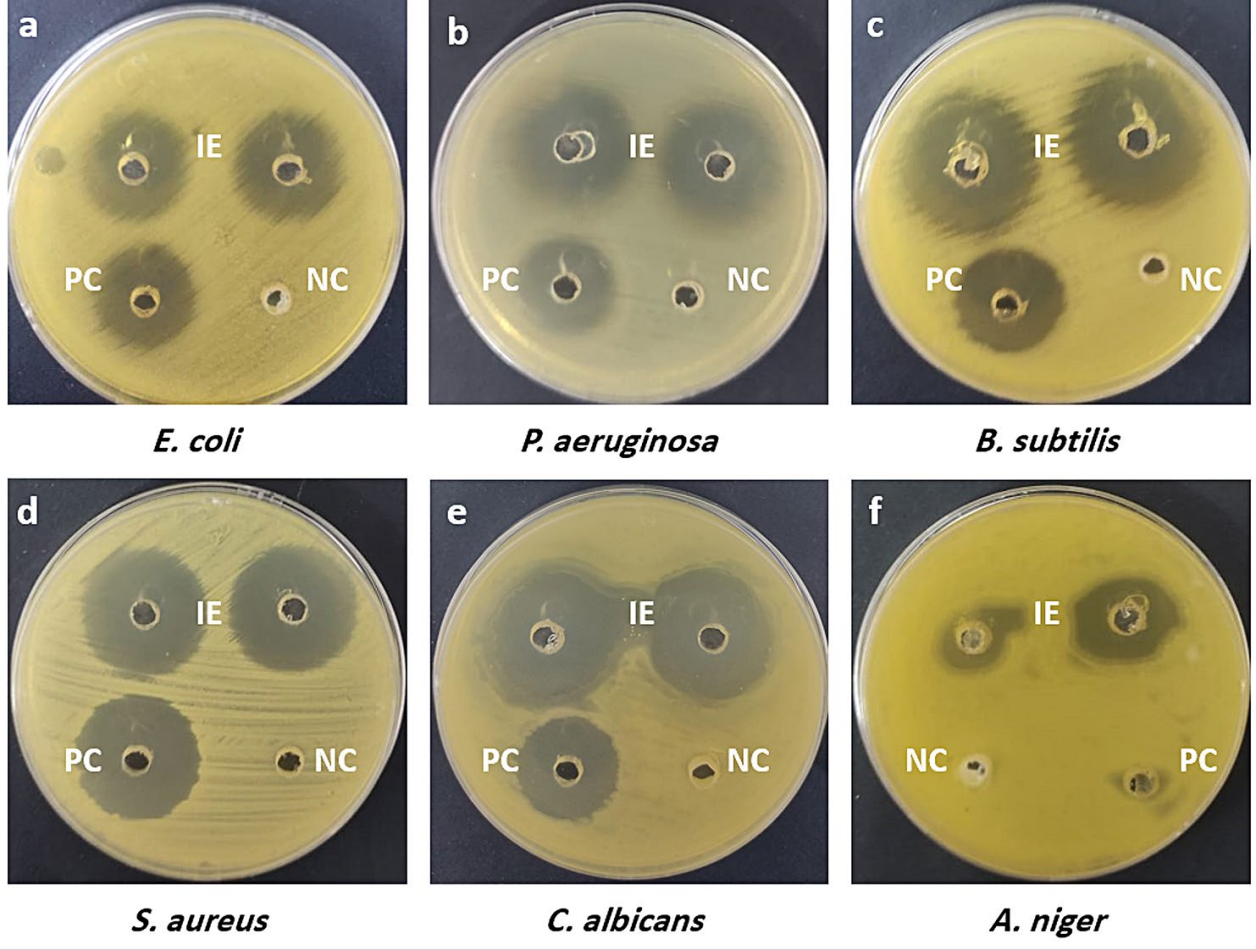
The inhibition zone (mm) of ethanolic ink extract (IE) of *S*. *pharaonis* at a concentration of 3 mg/mL against (**a**) *E. coli* (**b**) *P. aeruginosa* (**c**) *B. subtilis* (**d**) *S. aureus* (**e**) *C. albicans*, and (**f**) *A. niger*. PC Fluconazole and Gentamicin at a concentration of 10 mg/mL (positive control); NC 10% DMSO (negative control)

The antimicrobial efficacy of the ethanolic extract from *S*. *pharaonis* ink, at a concentration of 3 mg/mL, was initially assessed against various human pathogens including *E*. *coli*, *P*. *aeruginosa*, *B*. *subtilis*, *S*. *aureus*, *C*. *albicans*, and *A*. *niger* using the agar well diffusion method. The findings indicated significant suppression of growth for all tested pathogens. The experiment involved the measurement of inhibition zones, with corresponding data presented in Table 4; Fig. 6. Notably, the crude extract exhibited the largest zone of inhibition against *B*. *subtilis* (31.66 ± 1.15 mm), whereas the smallest zone was observed against *A*. *niger* (19.00 ± 1.00 mm). These inhibition zone results were compared with those of the positive controls, gentamicin and fluconazole.

**Table 4:** Antimicrobial activity (determined as inhibition zone diameter) of the ethanolic extract of *Sepia pharaonic* ink

Treatment	Diameter of inhibition zone (mm)					
	*E. coli*	*P. aeruginosa*	*B. subtilis*	*S. aureus*	*C. albicans*	*A. niger*
Ethanolic crud extract (3 mg/mL)	21.66 ± 0.58	23.00 ± 0.00	31.66 ± 1.15	29.66 ± 0.58	30.33 ± 2.30	19.00 ± 1.00
GM*	20.33 ± 0.58	20.00 ± 1.00	22.66 ± 0.58	23.33 ± 1.53		
FLZ*					25.33 ± 0.58	14.00 ± 0.00

(*) Positive control at the concentration of (10 mg/mL); GM, Gentamicin; FLZ, Fluconazole. The results represent the average diameter (mean ± SD) of triplicate tests

### MIC, MBC, and MFC of IE

The ethanolic extract derived from *S*. *pharaonis* ink underwent further assessment of its MIC, MBC, or MFC utilizing the microdilution assay, as detailed in Table 5. It exhibited activity against all tested pathogens including *E. coli*, *P. aeruginosa*, *B. subtilis*, *S. aureus*, *C. albicans*, and *A. niger*. MIC values of the ethanolic extract varied from 1.95 to 125 µg/mL. Particularly noteworthy was its strong activity against *C. albicans* with an MIC of 1.95 µg/mL, followed by *A*. *niger* with an MIC of 3.9 µg/mL, and *B*. *subtilis* with an MIC of 7.8 µg/mL. Furthermore, the MBC or MFC values of the extract ranged from 1.95 to 250 µg/mL.

**Table 5:** MIC, MBC and MFC of Sepia ink ethanolic extract against different human pathogens

Target pathogens	Extract concentration (µg/mL)	
	MIC	MBC or MFC
*E. coli*	125	250
*P. aeruginosa*	125	250
*B. subtilis*	7.8	15.6
*S. aureus*	15.6	62.5
*C. albicans*	1.95	1.95
*A. niger*	3.9	3.9

### ADME prediction analysis of flavonoids and phenolics extracted from ***Sepia***ink

**Fig. 7:**
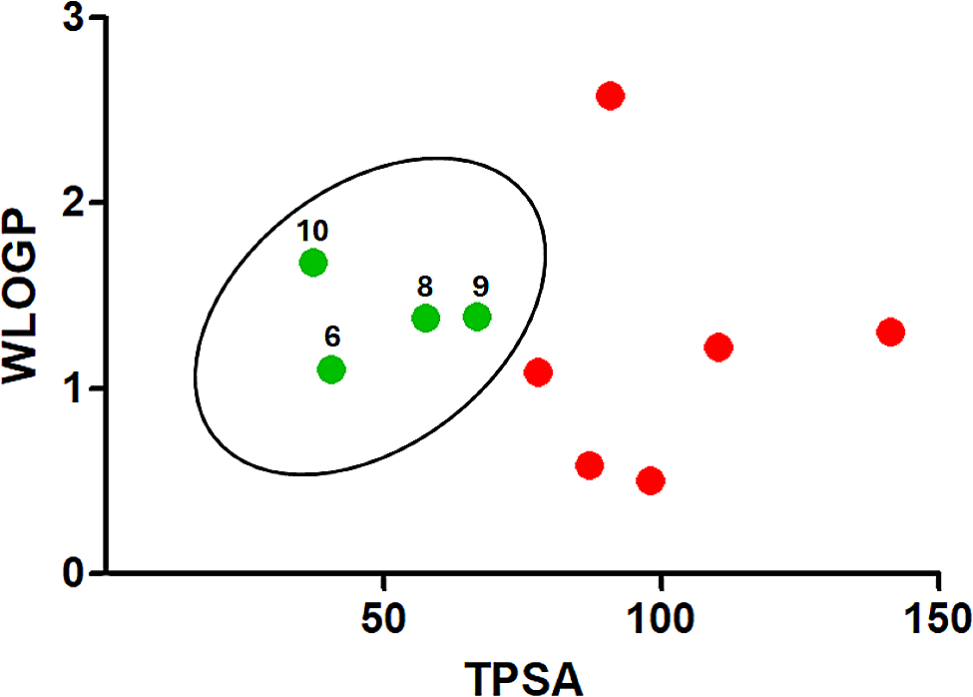
Boiled-Egg predictive model. Compounds with good evidence of brain access are represented by a green color (6, 8, 9, 10) while poorly absorbed molecules are represented by red color

**Fig. 8:**
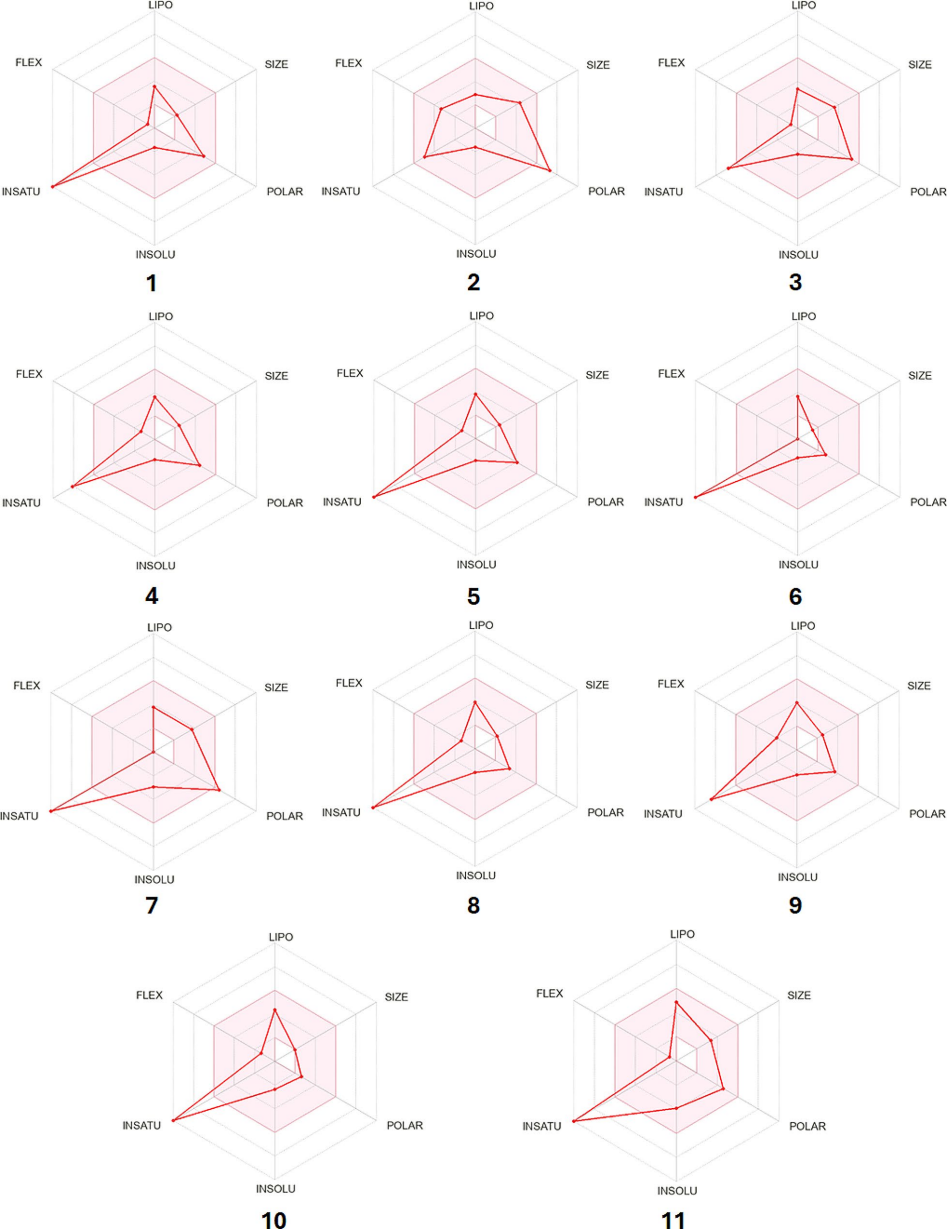
Bioavailability radar of bioactive drug-likeness compounds, where the pink areas are related to physicochemical properties (lipophilicity, molecular weight, solubility, and flexibility)

The pharmacokinetic properties of flavonoid and phenolic compounds were evaluated using the SwissADME online tool, and the findings were summarized in Table 6. All analyzed compounds adhere to Lipinski’s rule, with WLOGP values below 5.88 and TPSA below 131.6 Å2, indicating potential oral bioavailability. Based on pharmacokinetic parameters, all compounds except compound 2 demonstrate high gastrointestinal absorption. Additionally, compounds 6, 8, 9, and 10 exhibit the capability to access the blood-brain barrier (BBB), as shown in Fig. 7. Most compounds showed good bioavailability scores (0.55), except compound 2 (0.11). Drug-likeness can be further assessed using the bioavailability radar (Fig. 8). As shown in Fig. 8, most of the compounds fall entirely in the pink area which represents the optimal range for each physicochemical property (lipophilicity, flexibility, saturation, solubility, polarity, and size), and these compounds have the potential to be drug-like.

**Table 6:** Predicted pharmacokinetics properties of the flavonoids and phenolics-rich extract

Properties	Compounds										
	1	2	3	4	5	6	7	8	9	10	11
Physicochemical											
Mol. Wt. (g/mol)	170.12	354.31	290.27	184.15	180.16	110.11	302.19	164.16	194.18	148.16	270.24
Rotatable bonds	1	5	1	2	2	0	0	2	3	2	1
H-bond acceptors	5	9	6	5	4	2	8	3	4	2	5
H-bond donors	4	6	5	3	3	2	4	2	2	1	3
TPSA (Å^2^)	97.99	164.75	110.38	86.99	77.76	40.46	141.34	57.53	66.76	37.3	90.9
**Lipophilicity**											
WLOGP	0.5	-0.75	1.22	0.59	1.09	1.1	1.31	1.38	1.39	1.68	2.58
MLOGP	-0.16	-1.05	0.24	0.18	0.7	0.79	0.14	1.28	1	1.9	0.52
**Pharmacokinetics**											
GI absorption	High	Low	High	High	High	High	High	High	High	High	High
BBB permeant	No	No	No	No	No	Yes	No	Yes	Yes	Yes	No
**Drug-likeness**											
Lipinski’s violation	0	1	0	0	0	0	0	0	0	0	0
Bioavailability score	0.56	0.11	0.55	0.55	0.56	0.55	0.55	0.85	0.85	0.85	0.55

TPSA; molecular topological polar surface area; LogP; octanol-water partition coefficient; GI, gastrointestinal absorption; BBB, blood–brain barrier permeation

## Discussion

The ink of sepia is famous for its various therapeutic values. The crude ink extracts from various species have been recorded for their antimicrobial, antioxidant, anti-tumor, and many other properties [20, 48–51]. Extensive works have been conducted to find novel natural compounds with pharmaceutical activities and so the present work focused on the extraction of flavonoids from the ink of sepia pharaonic and the evaluation of its cytotoxic activities against some cancer cell lines and its antimicrobial activities against some bacterial and fungal strains. Cytotoxic activity of flavonoids and phenolics-rich extract was tested on four cancer cell lines: MCF7, Hep G2, A549, Caco2, and a normal cell line WI38 after 24 h. of treatment with different concentrations of the extract (0.0, 0.4, 1.6, 6.3, 25, 100 µg/mL) using MTT assay. These results indicated that the extract had a concentration-dependent inhibitory effect on the growth of all cell lines that were used and showed a significant cytotoxic effect at all concentrations (*P* < 0.0001). Cell viability (%) of these cell lines was investigated and the results showed that the A549 cancer cells were the most one that affected by the extract (IC_50_ = 2.8 µg/mL) followed by Hep G2 (IC_50_ = 7.1 µg/mL). However, its lower effect in the reduction of cell viability was recorded on MCF7 (IC_50_ = 21.6 µg/mL) followed by Caco2 (IC_50_ = 27.4 µg/mL). On the other hand, the normal cells (WI38) were weakly affected by the extract (The IC_50_ was recorded as 36.13 µg/mL). It has been shown that flavonoids inhibit the growth of many cancer cells, but not every polyphenolic compound has the same antiproliferative properties. They exhibit variations in their sensitivity and selectivity for tumor cells. This suggests that the cytotoxicity generated by flavonoids may be associated with specific cancer types. The sensitivities of cancer cells against flavonoids also can vary depending on the tissues from which they originated. Even when flavonoids have identical structures, compound-specific actions might influence distinct biochemical pathways, potentially having a differentially impactful effect on the development of certain neoplasms and pointing to tissue-specific cytotoxic action. In addition, variations in drug uptake, metabolism, and efflux mechanisms between different cell types can affect the intracellular concentration of the cytotoxic agent and thereby its effectiveness. These differences lead to different cellular and physiological response towards our treatment [52]. Response of the breast cancer cells MCF7 in the present study is in accordance with the results of Liu et al. [53] as they found that the proliferation of breast cancer MDA-MB-231 cells was suppressed in a concentration-dependent manner by using a combination of cisplatin (1, 2, 4, 8, 16 µg/mL) and polysaccharide that extracted from squid ink polysaccharide (SIP) (100, 200, 400, 800, 1600 µg/mL). The defatted ink of *Sepia pharaonic* exhibited a potent cytotoxic activity (69%) against MDA-MB-231 cells at the concentration of 1000 µg/mL) in the study of Abdel-Malek [50]. However, the study of Sasaki et al. [48] mentioned that the defatted ink lacks cytotoxicity against the tumor cells. It thus seems that the anti-tumor activity of the defatted ink was not due to direct cytotoxicity but to the enhancement of cellular immunity. Khudhair et al. [54] also studied the cytotoxic effect of the *Sepia prabahari* ink on the breast cancer cells, MCF7, and concluded that the crude ink showed anticancer activity and the melanin-free ink of the same species possessed a higher antiproliferative effect against this cell line. Hepatocellular carcinoma (Hep G2) was included in several works in this field, the study of Chen et al. [55] observed that the squid ink polysaccharides (SIP) didn’t show any effects on the proliferation of these cells, but inhibited invasion and cell migration. The crude and partially purified ink of the squid *Loligo duvauceli* was also studied by Diaz et al. [56] and showed cytotoxic activity against Hep G2 cell lines with a percentage of toxicity varied between 59% and 67%. Protein-fractionated ink of cuttlefish species also showed potent anticancer activity against Hep G2 cancer cell lines in the observations of Jesy et al. [57]. Cephalopod ink was previously reported in several studies for its antibiotic effects [31]. The ink of cuttlefish *Sepia pharaonic* in the present study was examined for its antimicrobial activities and screened against bacterial and fungal strains such as *E. coli*, *P. aeruginosa*, *B. subtilis*, *S. aureus*, *C. albicans*, and *A. niger*. The ethanolic extract of *Sepia* ink exhibited a promising antimicrobial activity against all tested pathogens and this could be attributed to the bioactive flavonoid compounds of the extract. The study of Rajaganapathi et al. [20] showed that the methanolic extracts of the ink of several species of cephalopoda: *Loligo duvaucelii*,* Sepia pharaonic*, *Sepiella inermis*, and *Octopus dollfusi* possess antibacterial activity against several bacterial strains. The Inhibition zone achieved by the ink of *L.duvaucelii* was recorded as > 3 mm against all tested bacteria in this study except *Proteus mirabilis* and *P. vulgaris*. The ink of Indian squids *L. duvauceli* was also studied by Nirmale et al. [58] for its antibacterial effect against some bacterial strains and they found that *Escherichia coli* exhibits a large inhibition zone despite it being the most sensitive one, followed by the *Salmonella* sp. strain and *V. cholerae.* The *Staphylococcus* sp. and *Micrococcus* sp. were also positively affected, but the inhibited zones that were recorded were smaller. Ink of the cuttlefish *S. pharaonis* was studied by Nithya and Ambikapathy [28] for their antibacterial effects against some human pathogens such as *Pseudomonas aeruginosa*, *K. pneumonia*, *E. coli*, and *Staphylococcus epidermidis* and showed a maximum inhibitory effect with diethyl ether. Girija et al. [29] characterized a novel antimicrobial protein from the ink of *Loligo duvauceli* and it showed that Lolduvin-S possesses high antibacterial activity against the drug-resistant test strains extended spectrum beta-lactamase (ESBL) producing strains of *E.coli* and *K. pneumoniae*, methicillin-resistant *S. aureus* (MRSA) and Amphotericin B resistant *C. albicans*; the size of inhibition zone ranges from a mean value of 20 mm for *E.coli* (ESBL), 21 mm for *K. pneumoniae* (ESBL), 22 mm for *S. aureus* (MRSA) and 20 mm for *C. albicans.* The methanolic ink extract of *Sepiella inermis* was examined by Vasantharaja et al. [59] on several bacterial and fungal strains and showed a significant antimicrobial effect. The maximum inhibited zone was recorded as 20 mm with *Candida albicans* and 19 mm with *Proteus vulgaris.* However, the reminder bacterial strains showed an inhibited zone ranging between 12 and 14 mm. Their study explained that this extract can inhibit the growth of both gram-positive and gram-negative pathogenic bacteria and fungi, indicating its wide spectrum of antimicrobial property so, they agree with the study of Abdel-Malek [50] in which the methanol extract of the defatted ink of the cuttlefishes *Sepia pharaonis* showed trace antibacterial activity against the bacterial strain *Staphylococcus aureus* with inhibited zone 8 mm. Bacterial pathogens isolated from carious dentine (*Lactobacillus acidophilus*, *Streptococcus mutans*, *Actinomyces viscosus*, and *Candida albicans*). Girija et al. [30] found that hexane ink extract from the Indian squid species showed high antimicrobial activity against four bacterial pathogens isolated from carious dentine with a mean zone of 18.33 mm for *Lactobacillus acidophillus*, 18.23 mm for *Candida albicans*, 14.46 mm for *Actinomyces viscosus* and 15.2 mm for *Streptococcus mutans*. However, the chloroform and acetone extract showed a medium activity. Di-ethyl ether showed trace activity against *L. acidophilus* and *C. albicans* and no activity against *S. mutans* and *A. viscosus*. Ethyl acetate extracts didn’t show any activity against any of the test pathogens under study.

The ink of *L. duvauceli* and *S. pharaonic* were studied for their antimicrobial activities by Diaz and Thilaga [31] against eight human pathogens. They found that the crude ink of *L. duvauceli* at 100 µl showed the highest activity against *Staphylococcus aureus* and the crude ink of *S. pharaonis* (100 µl) showed the highest activity against *V. fischeri* and *A. hydrophila*, while the minimum inhibition zone was recorded against *S. aureus*. Suja and Nisha [60] examined the antibacterial effect of methanol and water extract from *Loligo duvauceli* ink against two bacterial strains *E. coli* and *S. aureus*. Their study showed that the highest inhibition zone was recorded with the water extract (20 mm for *E. coli* and 15 mm for *S. aureus*). However, the water extract of the ink of *Sepia pharaonis* in the study of Abdel-Malek [50] didn’t exhibit any antibacterial activity on *E. coli* and *S. aureus* while the ink methanol extract exhibited trace activity against *S. aureus*. The study of Ismail and Riad [61] included the examination of the ink of *Sepia officinalis* for its antimicrobial activity on *Bacillus subtilis*, *Staphylococcus aureus*,* Escherichia coli*, *Pseudomonas aeruginosa*, *Salmonella typhi*, *Vibrio cholera*, and its antifungal pathogenic strains; *Aspergillus fumigatus* and *Candida albicans*. They reported that the aqueous extract has no antibacterial activity against all tested microbial species, while the highest antibacterial activity was detected in ink methanol extract against *P. aeruginosa.*

Lipinski’s rule is one of the essential bioinformatics tools for rational drug design and determining drug-likeness. This rule was used to evaluate the pharmacokinetic properties of the identified flavonoids and phenolics from IE. According to Lipinski’s “Rule of Five”, compounds should satisfy three out of the following five criteria to be considered potential drugs: (1) molecular weight not more than 500 Da; (2) maximum of 10 hydrogen bond acceptors; (3) maximum of 5 hydrogen bond donors; (4) the number of rotatable bonds not less than 10; (5) log P value (lipophilicity) not greater than 4.15. Additionally, for an orally available medication, WLOGP should be < 5.88 and TPSA < 131.6 Å^2^ [62]. Using the SwissADME online tool, we predicted the pharmacokinetic properties of flavonoid and phenolic compounds (Table 6). Most of these compounds are likely to be orally active drugs as they satisfy Lipinski’s criteria and have WLOGP values below 5.88 and TPSA below 131.6 Å^2^.

## Conclusion

In conclusion, the findings of this study demonstrate the potent cytotoxic and antimicrobial properties of the crude ethanolic extract derived from *S. pharaonis* ink. The extract exhibited significant cytotoxic effects against multiple cancer cell lines, notably A549 and Hep G2, with promising IC_50_ values. Mechanistic analyses revealed distinct cell cycle arrest patterns in various cancer cell lines, shedding light on potential pathways for further exploration. Additionally, the extract displayed remarkable antimicrobial activity against a range of human microbial pathogens, with particularly strong inhibition observed against *Candida albicans* ATCC 10,221. These results underscore the potential of *S. pharaonis* ink extract as a valuable source of bioactive compounds for further investigation in the development of novel anticancer and antimicrobial agents. Further studies are needed to elucidate the specific bioactive constituents responsible for these observed effects and to explore their therapeutic potential in depth.

### Electronic supplementary material

Below is the link to the electronic supplementary material.

Supplementary Material 1

## Data Availability

All data analyzed or generated for the research are included in the article.

## Abbreviations

MIC: Minimum Inhibitory Concentration
AD: Alzheimer’s disease
IE: Ink Extract
MCF7: Breast cancer cell line
Hep G2: Liver cancer cell line
A549: Lung cancer cell line
Caco2: Colon cancer cell line
WI38: Normal cell line
FBS: Fetal Bovine Serum
MTT assay: Tetrazolium dye assay
PBS: Phosphate Buffer Saline
V-FITC: V-fluorescein isothiocyanate
PI: Propidium Iodide
LB: Luria-Bertani
SDA: Sabouraud dextrose agar
OD: Optical Density
MBC: Minimum bactericidal concentration
MFC: Minimum fungicidal concentration
HPLC: High Performance Liquid Chromatography
SIP: squid ink polysaccharide
ESBL: extended spectrum beta-lactamase
MRSA: methicillin-resistant S. aureus
