# Supplementary material for: Antimicrobial and cytotoxic activities of flavonoid and phenolics extracted from Sepia pharaonis ink (Mollusca: Cephalopoda)
Source: BMC Biotechnol. 2024 Aug 12;24:54. doi: 10.1186/s12896-024-00880-3 (PMC11318128; doi:10.1186/s12896-024-00880-3)
Supplement: Supplementary file 1 — Supplementary Material 1 [file 12896_2024_880_MOESM1_ESM.pdf]

Antimicrobial and cytotoxic activities of flavonoid and phenolics extracted from *Sepia pharaonis* ink (Mollusca: Cephalopoda)

Authors and affiliations:

Asmaa R. Abdel- Malek[1], Alaa Y. Moustafa[2], Shimaa H. Salem[3*]

[1] Zoology and Entomology Department, Faculty of Science, Assiut University, 71526, Assiut, Egypt. (asmaa_a@aun.edu.eg, ORCID: **0000-0001-9249-4113**)

[2] Zoology Department, Faculty of Science, Sohag University, 82524 Sohag, Egypt.

[3] Botany and Microbiology Department, Faculty of Science, Assiut University, 71526, Assiut, Egypt

[*] Corresponding author at Department of Botany and Microbiology, Faculty of Science, Assiut University, Assiut, Egypt 71526. Tel: +201067657884

E-mail: shimaa.hassan@aun.edu.eg (Shimaa H. Salem, PhD).

ORCID ID: 0000-0002-2965-8143

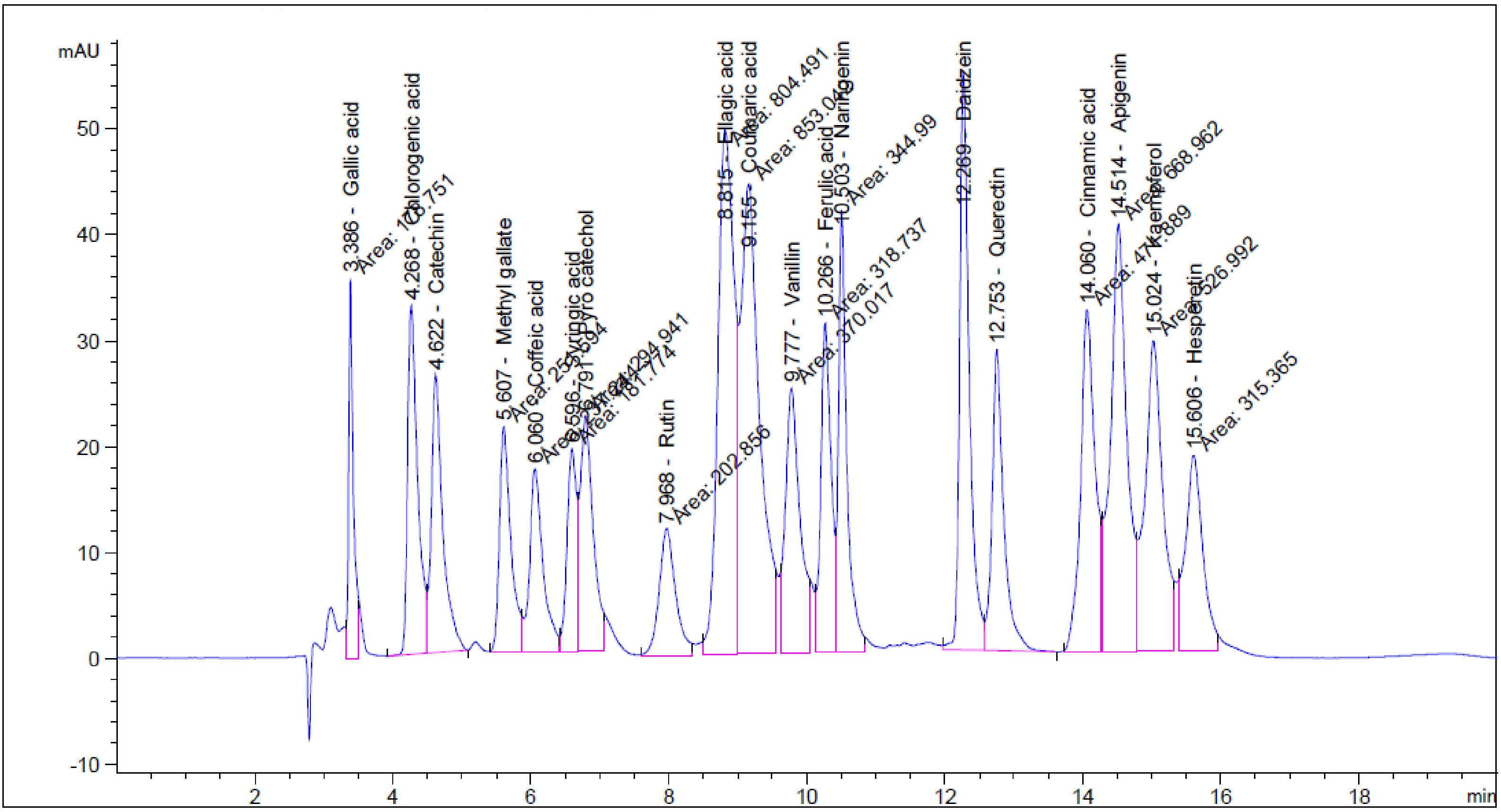

Fig. S1: HPLC chromatogram of standard flavonoids and phenolics.
